# Supplementary material for: Psychometric properties of a modified health belief model for cervical cancer and visual inspection with acetic acid among healthcare professionals in Ethiopia
Source: PLoS One. 2024 Apr 11;19(4):e0295905. doi: 10.1371/journal.pone.0295905 (PMC11008815; doi:10.1371/journal.pone.0295905)
Supplement: S2 Checklist — (DOCX) [file pone.0295905.s002.docx]

STROBE Statement—checklist of items that should be included in reports of observational studies

|  | Item No. | Recommendation | Page  No. | Relevant text from manuscript |
| --- | --- | --- | --- | --- |
| **Title and abstract** | 1 | (*a*) Indicate the study’s design with a commonly used term in the title or the abstract |  | Psychometric testing (exploratory and confirmatory factor analysis) |
|  |  | (*b*) Provide in the abstract an informative and balanced summary of what was done and what was found | 2 | Indicated in the abstract. |
| Introduction | | | |  |
| Background/rationale | 2 | Explain the scientific background and rationale for the investigation being reported | 3-5 | The tool has been widely used but not with the healthcare professional and with VIA. Choose VIA because it is widely used screening mechanism in Ethiopia to screen cervical cancer |
| Objectives | 3 | State specific objectives, including any prespecified hypotheses | 5 | To evaluate and validate the psychometric properties of the HBM constructs among professionals |
| Methods | | | |  |
| Study design | 4 | Present key elements of study design early in the paper | 5 | Cross sectional |
| Setting | 5 | Describe the setting, locations, and relevant dates, including periods of recruitment, exposure, follow-up, and data collection | 5 | Study was conducted in Addis Ababa University College of Health Sciences. The university has the largest teaching hospital under which accommodates many disciplines and faculties in health. |
| Participants | 6 | (*a*) *Cohort study*—Give the eligibility criteria, and the sources and methods of selection of participants. Describe methods of follow-up  *Case-control study*—Give the eligibility criteria, and the sources and methods of case ascertainment and control selection. Give the rationale for the choice of cases and controls  *Cross-sectional study*—Give the eligibility criteria, and the sources and methods of selection of participants | 5 | healthcare professionals aged 21- 65 years, no prior history of cervical cancer, speaks English and employed full-time |
|  |  | (*b*) *Cohort study*—For matched studies, give matching criteria and number of exposed and unexposed  *Case-control study*—For matched studies, give matching criteria and the number of controls per case |  | - |
| Variables | 7 | Clearly define all outcomes, exposures, predictors, potential confounders, and effect modifiers. Give diagnostic criteria, if applicable |  | Tool validation was outcome for this study |
| Data sources/ measurement | 8* | For each variable of interest, give sources of data and details of methods of assessment (measurement). Describe comparability of assessment methods if there is more than one group | *8* | *-* |
| Bias | 9 | Describe any efforts to address potential sources of bias |  | Participants were randomly selected |
| Study size | 10 | Explain how the study size was arrived at | 6 | In the current study a 5:1 ratio of participants to items was used to perform exploratory factor analysis (EFA). The modified HBM scale to be tested included 42 items, bringing the required number of participants to 210. Given the 24 items in the final modified version presented here the 5:1 ratio increased to 8.75:1 given |

Continued on next page

| Quantitative variables | 11 | Explain how quantitative variables were handled in the analyses. If applicable, describe which groupings were chosen and why |  | Variables were handled with their original nature. |
| --- | --- | --- | --- | --- |
| Statistical methods | 12 | (*a*) Describe all statistical methods, including those used to control for confounding | 7-8 | With all the criteria and assumption before running EFA; and each steps taken are noted. On top CFA was conducted and clearly showed the model fit indices was shown. |
|  |  | (*b*) Describe any methods used to examine subgroups and interactions |  | Not applicable |
|  |  | (*c*) Explain how missing data were addressed |  | No missing data: variables were checked for outliers based on the Mahalanobis distance and 16 cases with p< 0.001 were removed; remained data for analysis to be 194. |
|  |  | (*d*) *Cohort study*—If applicable, explain how loss to follow-up was addressed  *Case-control study*—If applicable, explain how matching of cases and controls was addressed  *Cross-sectional study*—If applicable, describe analytical methods taking account of sampling strategy | 6 | 5:1 ratio of sample size assumption was considered |
|  |  | (*e*) Describe any sensitivity analyses |  | Not applicable |
| Results | | | | |
| Participants | 13* | (a) Report numbers of individuals at each stage of study—eg numbers potentially eligible, examined for eligibility, confirmed eligible, included in the study, completing follow-up, and analysed |  | Data was collected for 210 of study participants and data were entered to 210 sample. But analysis was done for 194 participants as 16 cases were confirmed as outliers. |
|  |  | (b) Give reasons for non-participation at each stage |  | NONE |
|  |  | (c) Consider use of a flow diagram |  | Deliberately withhold creating a flow diagram as steps followed are few. |
| Descriptive data | 14* | (a) Give characteristics of study participants (eg demographic, clinical, social) and information on exposures and potential confounders | 10 | Indicated in Table 1 |
|  |  | (b) Indicate number of participants with missing data for each variable of interest |  | No missing data |
|  |  | (c) *Cohort study*—Summarise follow-up time (eg, average and total amount) |  | - |
| Outcome data | 15* | *Cohort study*—Report numbers of outcome events or summary measures over time |  | *-* |
|  |  | *Case-control study—*Report numbers in each exposure category, or summary measures of exposure |  | *-* |
|  |  | *Cross-sectional study—*Report numbers of outcome events or summary measures |  | *EFA and CFA conducted and as summary 6 factors emerged and from 42 item 24 remained in the CFA.* |
| Main results | 16 | (*a*) Give unadjusted estimates and, if applicable, confounder-adjusted estimates and their precision (eg, 95% confidence interval). Make clear which confounders were adjusted for and why they were included |  | - |
|  |  | (*b*) Report category boundaries when continuous variables were categorized |  | Socio demographic characters were categorized like age, work experience and income. Categorization was based on Ethiopian Demographic Health Survey and Human resource of the university to categorize the income. |
|  |  | (*c*) If relevant, consider translating estimates of relative risk into absolute risk for a meaningful time period |  | - |

Continued on next page

| Other analyses | 17 | Report other analyses done—eg analyses of subgroups and interactions, and sensitivity analyses |  | - |
| --- | --- | --- | --- | --- |
| Discussion | | | | |
| Key results | 18 | Summarise key results with reference to study objectives | 16 | Factors loaded, variance explained and model fit indices precisely summarized. |
| Limitations | 19 | Discuss limitations of the study, taking into account sources of potential bias or imprecision. Discuss both direction and magnitude of any potential bias | 19 | Due to of the cross-sectional nature of the data used, the stability of the initial and revised dimension scores over time were not examined. |
| Interpretation | 20 | Give a cautious overall interpretation of results considering objectives, limitations, multiplicity of analyses, results from similar studies, and other relevant evidence | 16-18 | Results were interpreted as per similarity and opposing findings are also discussed |
| Generalisability | 21 | Discuss the generalisability (external validity) of the study results | 19 | data for the present instrument revision were provided by a sample from a specific population, healthcare professionals; hence, the generalizability of the HBM instrument should be examined in other populations of women in Ethiopia, such as those from rural areas and women who are less educated. |
| Other information | |  | | |
| Funding | 22 | Give the source of funding and the role of the funders for the present study and, if applicable, for the original study on which the present article is based |  | SIDA has funded but the role of funder was specified only to financial support. Letter of support is uploaded as a supplement file. |

*Give information separately for cases and controls in case-control studies and, if applicable, for exposed and unexposed groups in cohort and cross-sectional studies.

**Note:** An Explanation and Elaboration article discusses each checklist item and gives methodological background and published examples of transparent reporting. The STROBE checklist is best used in conjunction with this article (freely available on the Web sites of PLoS Medicine at http://www.plosmedicine.org/, Annals of Internal Medicine at http://www.annals.org/, and Epidemiology at http://www.epidem.com/). Information on the STROBE Initiative is available at www.strobe-statement.org.
