# Supplementary material for: Psychometric properties of a modified health belief model for cervical cancer and visual inspection with acetic acid among healthcare professionals in Ethiopia
Source: PLoS One. 2024 Apr 11;19(4):e0295905. doi: 10.1371/journal.pone.0295905 (PMC11008815; doi:10.1371/journal.pone.0295905)
Supplement: S1 File — (DOCX) [file pone.0295905.s003.docx]

**Information sheet and consent form**

**Dear respondent**

**Greeting!**

My name is __________________________ I am a member of the research team, as data collector for the study that will be conducted to determine ‘**Cervical Cancer Screening Intention among health care professionals working at the College of Health Science of Addis Ababa University: Using Psychometrically Tested Health Belief Model Clued by Trans-Theoretical Model: 2020**. The study is part of a PhD research requirement conducted by **Sr. Semarya Berhe Lemlem**, studying her PhD in Addis Ababa University, College of Health Science, School of Nursing and Midwifery, in collaboration with Emory University, Nell Hodgson Woodruff School of Nursing. The study contains self-administered questionnaires to explore the necessary information about the above-mentioned issues. Thus, you are invited to take part in this study by giving genuine responses to all structured questions. Your cooperation and willingness are greatly appreciated and helpful in achieving the aim of the research.

The following information describes the research study in which you are being asked to participate. Please read the information carefully. In the end, you will be asked to sign if you agree to participate.

**PURPOSE OF THE STUDY:**

You are being asked to participate in a research study. The purpose of this study is to examine the beliefs and intention of the health care professionals working at the College of Health Science have related to Cervical Cancer and screening. You are being asked to be in the study because you are a professional working at the college of health science in Addis Ababa University.

**PROCEDURES:**

If you agree to participate in the study, the researcher will ask you to answer an item questionnaire about Cervical Cancer and screening, demographic, clinical and personal behavior questionnaire. The questionnaire will take approximately 60 minutes, and you will need to answer only one time.

**RISKS:**

We do not expect that any harm will happen to you because of joining this study. If you feel tired/exhausted, due to the nature of your job, you can stop temporarily and continue at your convenience.

**BENEFITS:**

There will be no direct benefit to you from participating in this study. However, the information that you provide may help health care professionals to better understand how Cervical Cancer Screening can be promoted. In case of injury We do not anticipate that any harm will occur to you as a result of participation in this study. However, if any physical injury resulting from participation in this study should occur, we will provide you with medical treatment according to the current standards of care in Ethiopia.

**ALTERNATIVE:**

Your alternative is not to participate in the study.

**CONFIDENTIALITY:**

Your answers will be confidential. Your name will not appear in any document. You will be identified through a number, which will be assigned in the questionnaire that you will receive. The relationship between your name and your answers will not be possible to link, because your name is not required on the questionnaire. The questionnaire and the informed consents will be secured in the researcher’s office, in a locked cabinet.

**COMPENSATION:**

You will not be paid for your participation in this study.

**RIGHT TO DECLINE OR WITHDRAW:**

Your participation in this study is voluntary. You are free to refuse to participate in the study or withdraw your consent at any time during the study. Your withdrawal or lack of participation will not affect anything. I would also like to inform you that this study will be approved by Addis Ababa University, College of Health Sciences Ethical Review Board (IRB).

Addis Ababa University

College of Health Sciences,

Institutional Review Board

Tel; +251 11 8 96 13 96

Email: [chs.irb@.aau.edu.et](mailto:chs.irb@.aau.edu.et)

If you have question about the study, the address of the principal investigator is:

**CONTACT INFORMATION:**

Semarya Berhe (+251-911-01-03-47) will gladly answer any questions you may have concerning the purpose, procedures, and outcome of this project.

**Advisors**

Dr. Mitike Molla (+251-911-13-18-05)

**PARTICIPANT AGREEMENT:**

I have read the information in this consent form and agree to participate in this study. I have had the chance to ask any questions I have about this study, and they have been answered for me. I am entitled to a copy of this form after it has been read and signed. Based on this information, I voluntarily agree to take part in this study

_______________________________ ______________

**Signature of Participant Date**

**PART I: SOCIO-DEMOGRAPHIC CHARACTERISTICS**

Under this part independent variables such as age, religion, marital status, work experience, profession and income are going to be filled:

| **Question** | **Responses** |
| --- | --- |
| 1. **How old are you?** | ___________________ in years |
| 1. **Marital status** | - 1. Single   2. Married   3. Divorced   4. Widowed   5. Cohabited |
| 1. **Service in years** | ___________________ in years |
| 1. **Educational level** | - 1. Diploma   2. BSc   3. Masters   4. MD   5. PhD   6. Others, specify________________ |
| 1. **Monthly income** | In birr_____________________ |
| 1. **Professional stream** | - 1. Clinical   2. Academic |
| 1. **Professional title** | 1. Nurse 2. Midwife 3. Physician 4. Clinical laboratory 5. Pharmacist 6. Anesthetist 7. Other; Specify __________________   **__________** |
| 1. **Unit of work** | - 1. Medical Ward   2. Surgical ward   3. Oncology   4. Outpatient   5. Other, specify _______________________ |

**PART II: ADAPTED CHAMPION’S HEALTH BELIEF MODEL SCALE FOR CERVICAL CANCER AND SCREENING AND TURKISH HEALTH BELIEF MODEL SCALE FOR CERVICAL CANCER AND SCREENING**

The Health Belief Model Scale for Cervical Cancer and Screening has 42 items in 5 subscales: Benefits of Pap Smear Test and Health Motivation, Barriers to Pap Smear Test, Perceived Seriousness of Cervical Cancer, Susceptibility to Cervical Cancer, and Health Motivation. All the items of subscales have 5-point Likert-type response choices: “strongly disagree (scores 1 point) disagree (scores 2 point), neutral (scores 3 point), agree (scores 4 point) strongly agree (scores 5 points). Higher scores indicate stronger feelings regarding that construct. All scales are positively related to screening behavior except for barriers, which have a negative association.

| **Benefits of VIA and Health Motivation** | 1Strongly disagree | 2  Disagree | 3  Neutral | 4  Agree | 5 strongly agree |
| --- | --- | --- | --- | --- | --- |
| 1. Having regular VIA will help to find changes to the cervix, before they turn into cancer |  |  |  |  |  |
| 1. If cervical cancer was found at a regular VIA test its treatment would not be so bad |  |  |  |  |  |
| 1. I think that having a regular VIA is the best way for cervical cancer to be diagnosed early |  |  |  |  |  |
| 1. Having regular VIA will decrease my chances of dying from cervical cancer |  |  |  |  |  |
| 1. I want to discover health problems early |  |  |  |  |  |
| 1. Maintaining good health is extremely important to me |  |  |  |  |  |
| 1. I look for new information to improve my health |  |  |  |  |  |
| 1. I feel it is important to carry out activities which will improve my health |  |  |  |  |  |
| **Barriers to VIA** | 1Strongly disagree | 2  Disagree | 3  Neutral | 4  Agree | 5 strongly agree |
| 1. I am afraid to have VIA for fear of a bad result |  |  |  |  |  |
| 1. I am afraid to have a because I don’t know what will happen |  |  |  |  |  |
| 1. I don’t know where to go for a VIA |  |  |  |  |  |
| 1. I would be ashamed to lie on a gynecologic examination table and show my private parts to have a VIA |  |  |  |  |  |
| 1. Having a VIA takes too much time |  |  |  |  |  |
| 1. Having a VIA is too painful |  |  |  |  |  |
| 1. Health professionals doing VIA are rude to women |  |  |  |  |  |
| 1. I neglect or cannot remember to have a VIA regularly |  |  |  |  |  |
| 1. I have other problems more important than having VIA in my life |  |  |  |  |  |
| 1. I am too old to have a VIA regularly |  |  |  |  |  |
| 1. There is no health center close to my house to have a VIA |  |  |  |  |  |
| 1. If there is cervical cancer development in my destiny, having a VIA cannot prevent it |  |  |  |  |  |
| 1. I prefer a female doctor to conduct a VIA |  |  |  |  |  |
| 1. I will never have a VIA if I have to pay for it |  |  |  |  |  |
| 1. I do not have time to get VIA |  |  |  |  |  |
| 1. The VIA may move the intrauterine device |  |  |  |  |  |
| 1. My partner does not want me to get VIA |  |  |  |  |  |
| 1. It is difficult to get an appointment for VIA |  |  |  |  |  |
| **Perceived Seriousness of Cervical Cancer** | 1Strongly disagree | 2  Disagree | 3  Neutral | 4  Agree | 5 strongly agree |
| 1. The thought of cervical cancer scares me |  |  |  |  |  |
| 1. When I think about cervical cancer, my heart beats faster |  |  |  |  |  |
| 1. I am afraid to think about cervical cancer |  |  |  |  |  |
| 1. Problems I would experience with cervical cancer would last a long time |  |  |  |  |  |
| 1. Cervical cancer would threaten a relationship with my boyfriend, husband, or partner |  |  |  |  |  |
| 1. If I had cervical cancer my whole life would change |  |  |  |  |  |
| 1. If I developed cervical cancer, I would not live longer than 5 years |  |  |  |  |  |
| **Susceptibility to Cervical Cancer** | 1Strongly disagree | 2  Disagree | 3  Neutral | 4  Agree | 5 strongly agree |
| 1. It is likely that I will get cervical cancer in the future |  |  |  |  |  |
| 1. My chances of getting cervical cancer in the next few years are high |  |  |  |  |  |
| 1. I feel I will get cervical cancer some time during my life |  |  |  |  |  |
| **Health Motivation** | 1Strongly disagree | 2  Disagree | 3  Neutral | 4  Agree | 5 strongly agree |
| 1. I eat well balanced meals for my health |  |  |  |  |  |
| 1. I exercise at least 3 times a week for my health |  |  |  |  |  |
| 1. I have regular health check-ups even when I am not sick |  |  |  |  |  |
| **Self-efficacy** | 1Strongly disagree | 2  Disagree | 3  Neutral | 4  Agree | 5 strongly agree |
| 1. I feel capable of arranging to have a VIA test. |  |  |  |  |  |
| 1. I feel capable of getting a VIA test. |  |  |  |  |  |
| 1. I feel capable of managing any emotional distress caused by VIA test |  |  |  |  |  |

**THANK YOU!!**
