# Supplementary material for: Psychometric properties of a modified health belief model for cervical cancer and visual inspection with acetic acid among healthcare professionals in Ethiopia
Source: PLoS One. 2024 Apr 11;19(4):e0295905. doi: 10.1371/journal.pone.0295905 (PMC11008815; doi:10.1371/journal.pone.0295905)
Supplement: S2 File — (PDF) [file pone.0295905.s004.pdf]

ADDIS ABABA UNIVERSITY, COLLEGE OF HEALTH SCIENCES (IRB)
አዲስ አበባ ዩኒቨርስቲ ጤናሳይንስ ኮሌጅ
Institutional Review Board

ANNEX 3
Form AAUMF 03-008

IRB's Decision

Meeting No: 04/2020 Meeting Date: April 29,2020
Protocol number: 017/20/Nursing

**Protocol Title:** Cervical cancer screening intention among health care professionals working at the college of health sciences of Addis Ababa university: using psychometrically tested health belief model clued by trans-theoretical Model: 2020	
Principal Investigator:	Semarya Berhe
Institute:	College of Health Sciences, AAU
Elements Reviewed (AAUMF 01-008)	☑ Attached ☐ Not attached
Review of Revised Application ☐ Yes ☐ No	Date of Previous review:
Decision of the meeting:	☑Approved ☐Approved with Recommendation ☐Resubmission ☐ Disapproved

I. Elements approved-
1. Protocol Version No: 2
2. Protocol Version Date:
3. Informed consent Version No. 2
4. Informed Consent Version Date:

II. Obligations of the PI-
1. Should comply with the standard international & national scientific and ethical guidelines
2. All amendments and changes made in protocol and consent form needs IRB approval
3. The PI should report SAE within 10 days of the event
4. End of the study, including manuscripts and thesis works should be reported to the IRB
5. The PI should report non-compliance and unanticipated events

III. TO NERC ☐

Institution Review Board (IRB) Approval: Period from: May 18,2020 to May 17,2021 Follow up report expected in

3 Months ___ 6 months ___ 9 months ✔ one year____

Chairperson, IRB
Dr. Adamu Addissie

Signature
Date: 18/05/2020

Institutional Review Board Office (IRB) Faculty of Medicine ADDIS ABABA UNIVERSITY
አዲስ አበባ ዩኒቨርስቲ

Scanned by CamScanner
